# Supplementary material for: Improving data sharing and knowledge transfer via the Neuroelectrophysiology Analysis Ontology (NEAO)
Source: Sci Data. 2025 May 29;12:907. doi: 10.1038/s41597-025-05213-3 (PMC12122730; doi:10.1038/s41597-025-05213-3)
Supplement: Supplementary file 1 — Supplementary Information [file 41597_2025_5213_MOESM1_ESM.pdf]

Supplementary Information

Table of Contents

Supplementary Text 2

Table S1. Results of a SPARQL query identifying the path of files that stored power spectral density (PSD) analysis results 5

Table S2. Results of a SPARQL query identifying the path of files that stored interspike interval histogram (ISIH) analysis 6

Table S3. Results of a SPARQL query identifying the path of files that stored analysis results obtained from artificial data 7

Figure S1. Code to insert NEAO *hasInput*, *hasOutput* and *usesParameter* relationships based on the provenance information captured by Alpaca and existing annotations with NEAO classes 8

Figure S2. Code to insert NEAO software implementation details based on the information captured by Alpaca 9

Figure S3. Code to insert *hasOutput* relationships for function executions that had a container as output 10

Supplementary Text

These are the dictionaries used to add NEAO annotations to Python functions inside a script that executed a specific variant of an analysis. The dictionary is stored as the __*ontology*__ function attribute, and its elements are detailed in Figure 8 in the main text. Namespaces are the same as defined in Table 1 in the main text. Details on how Alpaca applies the semantic annotations to the captured provenance are described in the toolbox documentation (https://alpaca-prov.readthedocs.io/en/latest/reference/ontologies.html).

NEAO annotations inserted into functions of Analysis 1.1

```
elephant.signal_processing.butter
    function = "neao_steps:ApplyButterworthFilter",
    arguments = {'signal': "neao_data:TimeSeries",
                 'lowpass_frequency': "neao_params:LowPassFrequencyCutoff",
                 'highpass_frequency': "neao_params:HighPassFrequencyCutoff",
                 'order': "neao_params:FilterOrder"},
    returns = {0: "neao_data:TimeSeries"}

elephant.spectral.welch_psd
    function = "neao_steps:ComputePowerSpectralDensityWelch",
    arguments = {'signal': "neao_data:TimeSeries",
                 'frequency_resolution': "neao_params:FrequencyResolution",
                 'overlap': "neao_params:WindowOverlapFactor",
                 'window': "neao_params:WindowFunction"},
    returns = {1: "neao_data:PowerSpectralDensity"}

neo.AnalogSignal.downsample
    function = "neao_steps:ApplyDownsampling",
    arguments = {'self': "neao_data:TimeSeries",
                 'downsampling_factor': "neao_params:DownsampleFactor"},
    returns = {0: "neao_data:TimeSeries"}
```

NEAO annotations inserted into functions of Analysis 1.2

```
elephant.signal_processing.butter
    function = "neao_steps:ApplyButterworthFilter",
    arguments = {'signal': "neao_data:TimeSeries",
                 'lowpass_frequency': "neao_params:LowPassFrequencyCutoff",
                 'highpass_frequency': "neao_params:HighPassFrequencyCutoff",
                 'order': "neao_params:FilterOrder"},
    returns = {0: "neao_data:TimeSeries"}

elephant.spectral.multitaper_psd
    function = "neao_steps:ComputePowerSpectralDensityMultitaper",
    arguments = {'signal': "neao_data:TimeSeries",
                 'peak_resolution': "neao_params:PeakResolution"},
    returns = {1: "neao_data:PowerSpectralDensity"}

neo.AnalogSignal.downsample
    function = "neao_steps:ApplyDownsampling",
    arguments = {'self': "neao_data:TimeSeries",
                 'downsampling_factor': "neao_params:DownsampleFactor"},
    returns = {0: "neao_data:TimeSeries"}
```

NEAO annotations inserted into functions of Analysis 1.3

```
elephant.signal_processing.butter
    function = "neao_steps:ApplyButterworthFilter",
    arguments = {'signal': "neao_data:TimeSeries",
                 'lowpass_frequency': "neao_params:LowPassFrequencyCutoff",
                 'highpass_frequency': "neao_params:HighPassFrequencyCutoff",
                 'order': "neao_params:FilterOrder"},
    returns = {0: "neao_data:TimeSeries"}

scipy.signal.welch
    function = "neao_steps:ComputePowerSpectralDensityWelch",
    arguments = {'x': "neao_data:TimeSeries",
                 'nperseg': "neao_params:WindowLengthSamples",
                 'noverlap': "neao_params:WindowOverlapSamples",
                 'fs': "neao_params:SamplingFrequency",
                 'window': "neao_params:WindowFunction"},
    returns = {1: "neao_data:PowerSpectralDensity"}

neo.AnalogSignal.downsample
    function = "neao_steps:ApplyDownsampling",
    arguments = {'self': "neao_data:TimeSeries",
                 'downsampling_factor': "neao_params:DownsampleFactor"},
    returns = {0: "neao_data:TimeSeries"}
```

NEAO annotations inserted into functions of Analysis 2.1

```
elephant.spike_train_surrogates.dither_spikes
    function = "neao_steps:GenerateUniformSpikeDitheringSurrogate",
    arguments = {'spiketrain': "neao_data:SpikeTrain",
                 'dither': "neao_params:DitheringTime"},
    returns = {'*': "neao_data:SpikeTrainSurrogate"}

elephant.statistics.isi
    function = "neao_steps:ComputeInterspikeIntervals",
    returns = {0: "neao_data:InterspikeIntervals"}

__main__.isi_histogram
    function = "neao_steps:ComputeInterspikeIntervalHistogram",
    arguments = {'isi_times': "neao_data:InterspikeIntervals",
                 'bin_size': "neao_params:BinSize"},
    returns = {0: "neao_data:InterspikeIntervalHistogram"}

__main__.aggregate_isi_histograms
    function = "neao_steps:ApplySum",
    returns = {0: "neao_data:InterspikeIntervalHistogram"}

__main__.mean_and_sd
    function = ["neao_steps:ComputeMean",
                "neao_steps:ComputeStandardDeviation"],
    returns = {0: "neao_data:InterspikeIntervalHistogram",
               1: "neao_data:Data"}
```

NEAO annotations inserted into functions of Analysis 2.2

```
elephant.spike_train_surrogates.trial_shifting
    function = "neao_steps:GenerateTrialShiftingSurrogate",
    arguments = {'spiketrains': "neao_data:SpikeTrain",
                 'dither': "neao_params:DitheringTime"},
    returns = {'***': "neao_data:SpikeTrainSurrogate"}

elephant.statistics.isi
    function = "neao_steps:ComputeInterspikeIntervals",
    returns = {0: "neao_data:InterspikeIntervals"}

__main__.isi_histogram
    function = "neao_steps:ComputeInterspikeIntervalHistogram",
    arguments = {'isi_times': "neao_data:InterspikeIntervals",
                 'bin_size': "neao_params:BinSize"},
    returns = {0: "neao_data:InterspikeIntervalHistogram"}

__main__.aggregate_isi_histograms
    function = "neao_steps:ApplySum",
    returns = {0: "neao_data:InterspikeIntervalHistogram"}

__main__.mean_and_sd
    function = ["neao_steps:ComputeMean",
                "neao_steps:ComputeStandardDeviation"],
    returns = {0: "neao_data:InterspikeIntervalHistogram",
               1: "neao_data:Data"}
```

NEAO annotations inserted into functions of Analysis 3

```
elephant.spike_train_generation.homogeneous_poisson_process
    function = "neao_steps:GenerateStationaryPoissonProcess",
    arguments = {'rate': "neao_params:FiringRate"},
    returns = {0: "neao_data:SpikeTrain"}

elephant.spike_train_generation.homogeneous_gamma_process
    function = "neao_steps:GenerateStationaryGammaProcess",
    arguments = {'a': "neao_params:ShapeFactor",
                 'b': "neao_params:FiringRate"},
    returns = {0: "neao_data:SpikeTrain"}

elephant.statistics.isi
    function = "neao_steps:ComputeInterspikeIntervals",
    arguments = {'spiketrain': "neao_data:SpikeTrain"},
    returns = {0: "neao_data:InterspikeIntervals"}

elephant.statistics.cv2
    function = "neao_steps:ComputeCV2",
    arguments = {'time_intervals': "neao_data:InterspikeIntervals"},
    returns = {0: "neao_data:CV2"}

__main__.isi_histogram
    function = "neao_steps:ComputeInterspikeIntervalHistogram",
    arguments = {'isi_times': "neao_data:InterspikeIntervals",
                 'bin_size': "neao_params:BinSize"},
    returns = {0: "neao_data:InterspikeIntervalHistogram"}
```

File path
.../reach2grasp/psd_by_trial/i140703-001/1.png
.../reach2grasp/psd_by_trial/i140703-001/10.png
.../reach2grasp/psd_by_trial/i140703-001/100.png
.../reach2grasp/psd_by_trial/i140703-001/101.png
.../reach2grasp/psd_by_trial/i140703-001/102.png
.../reach2grasp/psd_by_trial/i140703-001/103.png
.../reach2grasp/psd_by_trial/i140703-001/104.png
.../reach2grasp/psd_by_trial/i140703-001/105.png
.../reach2grasp/psd_by_trial/i140703-001/106.png
.../reach2grasp/psd_by_trial/i140703-001/107.png
.../reach2grasp/psd_by_trial/i140703-001/108.png
.../reach2grasp/psd_by_trial/i140703-001/109.png
.../reach2grasp/psd_by_trial/i140703-001/11.png
.../reach2grasp/psd_by_trial/i140703-001/110.png
.../reach2grasp/psd_by_trial/i140703-001/111.png
(omitted 450 lines)
.../reach2grasp/psd_by_trial_3/i140703-001/86.png
.../reach2grasp/psd_by_trial_3/i140703-001/87.png
.../reach2grasp/psd_by_trial_3/i140703-001/88.png
.../reach2grasp/psd_by_trial_3/i140703-001/89.png
.../reach2grasp/psd_by_trial_3/i140703-001/9.png
.../reach2grasp/psd_by_trial_3/i140703-001/90.png
.../reach2grasp/psd_by_trial_3/i140703-001/91.png
.../reach2grasp/psd_by_trial_3/i140703-001/92.png
.../reach2grasp/psd_by_trial_3/i140703-001/93.png
.../reach2grasp/psd_by_trial_3/i140703-001/94.png
.../reach2grasp/psd_by_trial_3/i140703-001/95.png
.../reach2grasp/psd_by_trial_3/i140703-001/96.png
.../reach2grasp/psd_by_trial_3/i140703-001/97.png
.../reach2grasp/psd_by_trial_3/i140703-001/98.png
.../reach2grasp/psd_by_trial_3/i140703-001/99.png

Table S1: Results of a SPARQL query identifying the path of files that stored power spectral density (PSD) analysis results. This table was aggregated to produce summaries based on the root file path.

**File path**
.../isi_histograms/1.png
.../isi_histograms/10.png
.../isi_histograms/100.png
.../isi_histograms/101.png
.../isi_histograms/102.png
.../isi_histograms/103.png
.../isi_histograms/104.png
.../isi_histograms/105.png
.../isi_histograms/106.png
.../isi_histograms/107.png
.../isi_histograms/108.png
.../isi_histograms/109.png
.../isi_histograms/11.png
.../isi_histograms/110.png
.../isi_histograms/111.png
(omitted 182 lines)
.../isi_histograms/97.png
.../isi_histograms/98.png
.../isi_histograms/99.png
.../reach2grasp/surrogate_isih_1/i140703-001/Unit 15001.png
.../reach2grasp/surrogate_isih_1/i140703-001/Unit 36001.png
.../reach2grasp/surrogate_isih_1/i140703-001/Unit 48001.png
.../reach2grasp/surrogate_isih_1/i140703-001/Unit 59001.png
.../reach2grasp/surrogate_isih_1/i140703-001/Unit 6002.png
.../reach2grasp/surrogate_isih_1/i140703-001/Unit 7001.png
.../reach2grasp/surrogate_isih_2/i140703-001/Unit 15001.png
.../reach2grasp/surrogate_isih_2/i140703-001/Unit 36001.png
.../reach2grasp/surrogate_isih_2/i140703-001/Unit 48001.png
.../reach2grasp/surrogate_isih_2/i140703-001/Unit 59001.png
.../reach2grasp/surrogate_isih_2/i140703-001/Unit 6002.png
.../reach2grasp/surrogate_isih_2/i140703-001/Unit 7001.png

Table S2: Results of a SPARQL query identifying the path of files that stored interspike interval histogram (ISIH) analysis results. This table was aggregated to produce summaries based on the root file path.

File path
.../isi_histograms/1.png
.../isi_histograms/10.png
.../isi_histograms/100.png
.../isi_histograms/101.png
.../isi_histograms/102.png
.../isi_histograms/103.png
.../isi_histograms/104.png
.../isi_histograms/105.png
.../isi_histograms/106.png
.../isi_histograms/107.png
.../isi_histograms/108.png
.../isi_histograms/109.png
.../isi_histograms/11.png
.../isi_histograms/110.png
.../isi_histograms/111.png
(omitted 170 lines)
.../isi_histograms/86.png
.../isi_histograms/87.png
.../isi_histograms/88.png
.../isi_histograms/89.png
.../isi_histograms/9.png
.../isi_histograms/90.png
.../isi_histograms/91.png
.../isi_histograms/92.png
.../isi_histograms/93.png
.../isi_histograms/94.png
.../isi_histograms/95.png
.../isi_histograms/96.png
.../isi_histograms/97.png
.../isi_histograms/98.png
.../isi_histograms/99.png

Table S3: Results of a SPARQL query identifying the path of files that stored analysis results obtained from artificial data. This table was aggregated to produce summaries based on the root file path.

Figure S1: Code to insert NEAO *hasInput*, *hasOutput* and *usesParameter* relationships based on the provenance information captured by Alpaca and existing annotations with NEAO classes. For every node annotated with the NEAO *AnalysisStep* class, it checks if any of the PROV-O relationships *used* or *wasGeneratedBy* points to a node annotated with the NEAO Data class. If true, the corresponding *hasInput* or *hasOutput* relationship is inserted. Finally, if the node has an Alpaca *hasParameter* property pointing to a node annotated with the NEAO *AnalysisParameter* class, the corresponding NEAO *usesParameter* relationship is inserted.

```
PREFIX prov: <http://www.w3.org/ns/prov#>
PREFIX alpaca: <http://purl.org/alpaca#>
PREFIX rdf: <http://www.w3.org/1999/02/22-rdf-syntax-ns#>
PREFIX neao_base: <http://purl.org/neao/base#>

# Parameter triples
INSERT {
  ?function neao_base:usesParameter ?parameter .
}
WHERE {
  ?function rdf:type neao_base:AnalysisStep .
  ?function alpaca:hasParameter ?parameter .
  ?parameter rdf:type neao_base:AnalysisParameter .
};

# Input triples
INSERT {
  ?function neao_base:hasInput ?data .
}
WHERE {
  ?function rdf:type neao_base:AnalysisStep .
  ?function prov:used ?data .
  ?data rdf:type neao_base:Data .
};

# Output triples
INSERT {
  ?function neao_base:hasOutput ?data .
}
WHERE {
  ?function rdf:type neao_base:AnalysisStep .
  ?data prov:wasGeneratedBy ?function .
  ?data rdf:type neao_base:Data .
}
```

Figure S2: Code to insert NEAO software implementation details based on the information captured by Alpaca. For each function execution node (annotated with the Alpaca *FunctionExecution* and NEAO *AnalysisStep* classes), details are obtained from the *usedFunction* relationship (package information, function name, and version). The package string is derived from Alpaca's *implementedIn* property, excluding user-defined functions. The *functionName* and *functionVersion* properties provide the function name and version. The package Python name is mapped to a readable name (e.g., *SciPy* for *scipy*). For class methods, the actual function name is extracted by splitting the class name prefix by the dot. A NEAO *isImplementedIn* relationship is added to the function execution node, pointing to a node identified by a URI constructed from the package and function information and annotated with the NEAO *Function* class. A package node, identified by another URI and annotated with the NEAO *SoftwarePackage* class, is also added. Remaining NEAO properties describing the function (*nameInDefinition*, *isImplementedInPackage*) and package (*packageName*, *packageVersion*) are added to their respective nodes.

```
PREFIX alpaca: <http://purl.org/alpaca#>
PREFIX rdf: <http://www.w3.org/1999/02/22-rdf-syntax-ns#>
PREFIX neao_base: <http://purl.org/neao/base#>

INSERT {
  ?function_execution neao_base:isImplementedIn ?function_uri .
  ?function_uri rdf:type neao_base:Function .
  ?function_uri neao_base:nameInDefinition ?function_name_in_def .
  ?function_uri neao_base:isImplementedInPackage ?package_uri .
  ?package_uri rdf:type neao_base:SoftwarePackage .
  ?package_uri neao_base:packageName ?package_name .
  ?package_uri neao_base:packageVersion ?function_version .
}
WHERE {
{
  SELECT DISTINCT ?function_execution ?package_uri
                ?package_name ?function_version
                ?function_uri ?function_name_in_def WHERE {

    # Extract non user-defined functions
    ?function_execution rdf:type alpaca:FunctionExecution, neao_base:AnalysisStep .
    ?function_execution alpaca:usedFunction ?function_implementation .
    ?function_implementation alpaca:implementedIn ?package_str .
    FILTER(?package_str != "__main__") .

    # Extract function information
    ?function_implementation alpaca:functionName ?function_name_str .
    ?function_implementation alpaca:functionVersion ?function_version .

    # Extract package name
    BIND(STRBEFORE(?package_str, ".") AS ?package) .
    VALUES (?package ?package_name) {
      ("neo" "Neo")
      ("elephant" "Elephant")
      ("scipy" "SciPy")
    }

    # Extract function definition name
    BIND(IF(CONTAINS(?function_name_str, "."),
           STRAFTER(?function_name_str, "."),
           ?function_name_str) AS ?function_name_in_def) .

    # Define URIs
    BIND(CONCAT("urn:neao:Python:", ?package, ":", ?function_version) AS ?package_urn) .
    BIND(URI(?package_urn) AS ?package_uri) .
    BIND(URI(CONCAT(?package_urn, ":", ?function_name_str, ":", ?function_version))
        AS ?function_uri) .
  }
}}
```

Figure S3: Code to insert *hasOutput* relationships for function executions that had a container as output. The SPARQL code in Listing 1 misses function executions that output containers whose elements are data entities identified by annotations with the NEAO *Data* class. Therefore, for every function execution node annotated with the NEAO *AnalysisStep* class, each output is identified through the PROV-O *generated* relationship. If the output is a container node (annotated with PROV-O's *Collection* class), its members are checked for annotations with the NEAO *Data* class. The number of members with the NEAO class annotation is obtained. If greater than zero, the NEAO *hasOutput* relationship is added mapping the function execution node to the container.

```
PREFIX prov: <http://www.w3.org/ns/prov#>
PREFIX rdf: <http://www.w3.org/1999/02/22-rdf-syntax-ns#>
PREFIX neao_base: <http://purl.org/neao/base#>

# Insert outputs where steps generated containers with objects
# annotated with NEAO Data classes

INSERT {
  ?function neao_base:hasOutput ?output .
}
WHERE {
  ?function rdf:type neao_base:AnalysisStep .
  ?function prov:generated ?output .
  ?output rdf:type prov:Collection .
  {
    SELECT ?output (count(?data) AS ?n_data) WHERE {
      ?output prov:hadMember ?data .
      ?data rdf:type neao_base:Data .
    } GROUP BY ?output
  }
  FILTER (?n_data > 0) .
}
```
